# New-Onset Lichen Planus Induced by the Pfizer COVID-19 Vaccine

**DOI:** 10.1155/2022/2082445

**Published:** 2022-09-17

**Authors:** Fadi A. Alghamdi, Shahad T. Khayyat, Mohammed M. Alshareef, Wala'a Felemban

**Affiliations:** ^1^Department of Dermatology, King Fahad Armed Forces Hospital, Jeddah, Saudi Arabia; ^2^College of Medicine and Surgery, King Abdulaziz University, Jeddah, Saudi Arabia; ^3^Department of Dermatology, King Abdulaziz Hospital, Makkah, Saudi Arabia; ^4^Department of Pathology, King Fahad Armed Forces Hospital, Jeddah, Saudi Arabia

## Abstract

**Objective:**

Coronavirus disease 2019 (COVID-19) vaccine distribution continues to expand; however, increased cutaneous reactions have been reported. Several recent studies suggest a link between COVID-19 vaccination and the development of various cutaneous complications. Lichen planus is a chronic, immune-mediated, inflammatory dermatological illness with an unclear etiology. In this case report, we assessed the relationship between COVID-19 vaccination (Pfizer) and lichen planus diagnosis and evaluated the link between additional doses of the vaccine and disease progression.

**Methods:**

Complete clinical, laboratory, and histopathological assessment of a patient was performed with ethical and privacy considerations. Written informed consent for all clinical data, images, and publication was obtained from the patient.

**Results:**

New-onset lichen planus appeared 48 hours after the first dose of the Pfizer vaccine. The symptoms worsened following the second dose. The patient responded gradually to topical corticosteroids, and lichen planus was controlled within 21 days.

**Conclusion:**

Our case significantly contributes to the literature by highlighting that additional doses of the Pfizer vaccine can contribute to disease progression. Therefore, reporting the patient's condition associated with COVID-19 vaccination should be considered. Future studies should be performed to investigate the combined onset of lichen planus and multisystem COVID-19 vaccine-related complications.

## 1. Introduction

The coronavirus disease 2019 (COVID-19) pandemic was announced in March 2020 by the World Health Organization. Healthcare, social, and economic disturbances became widespread in most countries [1], encouraging further investigation and development of vaccines against severe acute respiratory syndrome coronavirus 2, as to date, no effective management existed [1]. As the COVID-19 vaccines became widely distributed, cutaneous reactions were increasingly observed. Previously, 414 registry individuals with dermatologic responses after receiving mRNA COVID-19 vaccines from Moderna (83%) and Pfizer (17%) were evaluated, of which systemic manifestations—including fatigue, headache, fever, and muscle and joint pain—were reported [2]. A wide range of documented skin conditions was identified following COVID-19 immunization, including local responses at the injection site, urticaria, and morbilliform eruptions with unexpected adverse events (including pityriasis-rosea-like rashes) [2]. Moreover, a case of lichen planus (LP) caused by COVID‐19 vaccination was reported [2]. The prevalence of LP, a mucocutaneous, inflammatory dermatosis [3] is approximately 1% in the United States. It mainly affects individuals aged 40–60 years; however, LP has also been identified in younger individuals, and males and females are equally affected [3]. Polygonal, flat-topped, violaceous papules and plaques are typical characteristics of LP. Physical assessment of oral or cutaneous LP may reveal “Wickham's striae,” superimposed, reticulated white scales [3]. The clinical findings based on lesion morphology include hypertrophic, bullous, actinic, and erosive types of LP. Hypertrophic LP, defined as scaly, hyperkeratotic, pruritic flat-topped plaques, are frequently found on the anterior distal lower limbs. Bullous LP appears as vesicles or bullae inside LP lesions. Actinic LP is characterized as atrophic, hyperpigmented lesions with a rolling edge that occur in sun-exposed areas and are suspected to be caused by ultraviolet radiation. Erosive LP refers to painful, eroded, or ulcerated lesions that usually include mucosal tissues and can cause scarring. The appearance of violaceous plaques with central clearing and atrophy is the characteristic of annular atrophic LP, an uncommon variant of LP. LP pigmentosus is described as hyperpigmented, lichenoid plaques distributed in sun-exposed or flexural regions [3].

Topical corticosteroids of medium to high potency are used as a first-line treatment for cutaneous LP. Based on the severity of the condition and the physician's expertise, other methods, such as intralesional injection, may be applied [3]. As a second-line therapy, systemic corticosteroids (oral or intramuscular injection) are commonly administered. Oral retinoid and phototherapy are other treatment options. Additional medications are being evaluated to treat this illness, including griseofulvin, oral metronidazole, efalizumab, thalidomide, sulfasalazine, mycophenolate mofetil, ciclosporin, and azathioprine [3].

While the correlation between LP and COVID-19 vaccinations should be determined, no studies investigating this relationship exist, making case reports a valuable source of information on the influence of additional vaccine doses on disease progression.

Thus, this case is the first to report new-onset LP following Pfizer vaccine administration, demonstrating a positive correlation between subsequent doses of the Pfizer vaccine and the progression of LP.

## 2. Case Presentation

A 67-year-old woman with hypertension, diabetes mellitus, and hypercholesterolemia presented to our dermatology outpatient clinic with chief complaints of multiple, new-onset, red to purple, itchy skin lesions after receiving her first dose of the Pfizer COVID-19 vaccine 5 months before attending our clinic. Skin lesions initially appeared on her back 2 days after vaccination. She received the second Pfizer vaccine dose after 30 days and gradually developed a new and similar skin manifestations associated with pruritus over the bilateral shoulders and chest. There was no bloody or purulent discharge observed, and no similar previous episodes had occurred. The patient was a nonsmoker, and when queried about possible provoking factors, she confirmed that there was no evidence of infection or alteration in medication, no recent social or emotional imbalances, negative history of food or drug allergies, and no family history of similar complaints.

**Figure 1:**
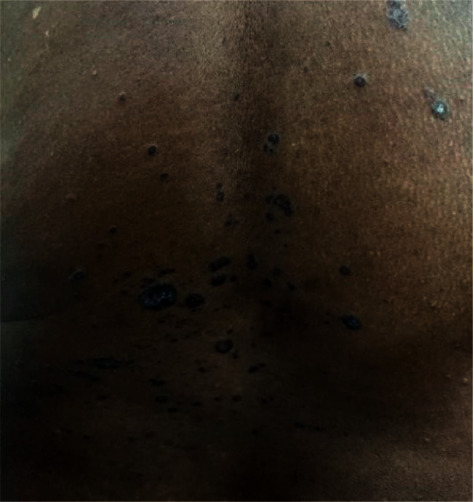
Multiple annular, hypertrophic, violaceous, well-demarcated papules and plaques over the back.

**Figure 2:**
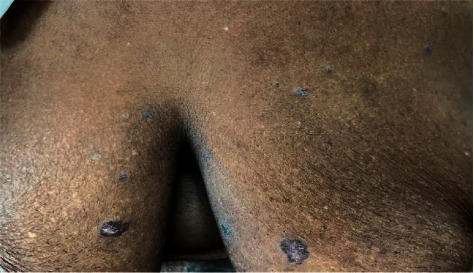
Multiple annular, hypertrophic, violaceous, well-demarcated papules and plaques on the chest.

Physical examination showed multiple annular, hypertrophic, scaly, violaceous, and well-demarcated polygonal papules and plagues on her back (Figure 1), chest (Figure 2), and bilateral shoulders. No scalp, oral, genital, or nail involvement was noted. Hepatitis serology showed negative results. A papule biopsy on her back was performed, revealing a separated epidermis from the underlying dermis; hyperkeratosis, hypergranulosis, and superficial, dermal, band-like lymphocytic infiltrates were observed. Dysplasia and malignancy were not evident. Considering these factors and test results, we diagnosed the patient with LP.

**Figure 3:**
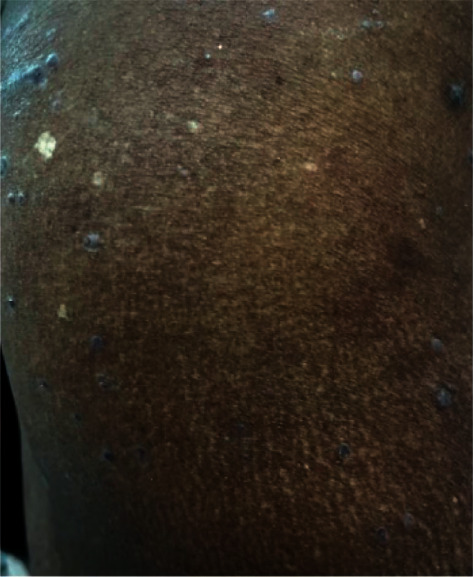
Improvement of the lichen planus lesions over the shoulders.

The patient was followed up after 14 days (Figure 3), during which she gradually responded to topical corticosteroids without new active skin lesions developing, and the disease was controlled within 21 days.

Informed written consent for the publication of all patient photographs and medical information was provided to the authors at the time of article submission to the journal, with the understanding that this information may be publicly available. The study was approved by the responsible research and ethics committee at King Fahd Armed Forces Hospital (approval number: REC 489).

## 3. Discussion

COVID-19 immunizations and related adverse effects are new to the medical field; therefore, early detection and management of rare COVID-19 vaccination-related dermatological cases and consideration of the third vaccination dose are vital.

Our patient developed new-onset cutaneous LP 2 days after receiving the first Pfizer dose, and progression occurred following the second dose. Another case of cutaneous LP was reported within 2 days; however, symptoms appeared after the second Pfizer dose [4]. Conversely, another case of cutaneous LP related to the Pfizer vaccine occurred 2 weeks after the second dose [5]. Two other patients were previously observed. The first patient suffered a relapse of lichen planopilaris 14 days after the second AstraZeneca vaccine dose, and the second suffered an LP flare-up 3 days after the first Sinopharm vaccine dose [6]. All previously mentioned cases [4–6] included middle-aged women, similar to our case. However, these participants had a controlled LP that was provoked by the COVID-19 vaccine [4–6].

LP is an inflammatory disorder of the skin, hair, nails, and mucous membranes; while its pathogenesis is not fully understood, it represents a T-cell-mediated autoimmune disease [3]. In addition, this condition is associated with previous exposure to triggering agents, including viruses, vaccines, and drugs [3].

In July 2020, a patient tested positive for COVID-19 and was diagnosed with annular LP 5 days later [7]. The author of that study concluded that LP appeared to directly correlate with the positivity rate of hepatitis C in a given population, in addition to vaccines for hepatitis B, influenza, rabies, diphtheria, tetanus, pertussis, measles, mumps, and rubella [7]. Clinical experiments showed that these vaccines activate a Th1 response, increasing interleukin 2, tumor necrosis factor (TNF), and interferon (IFN) serum levels (cytokines that play a role in LP development) [8]. Although the pathogenesis remains unknown, upregulation of the Th1 response increases the levels of proapoptotic cytokines, such as TNF*α* and IFN*γ*, which have been identified as critical factors for basal keratinocyte apoptosis in LP [3]. Furthermore, antimalarials, angiotensin-converting enzyme inhibitors, thiazide diuretics, nonsteroidal anti-inflammatory drugs, quinidine, beta-blockers, TNF*α* inhibitors, and gold are among the medications linked to LP [9, 10]. The LP onset in our patient might be attributable to the COVID-19 vaccine, as the first episode of cutaneous symptoms emerged after receiving the first Pfizer vaccine dose. Moreover, the patient confirmed that she had progressively developed multiple, new, similar pruritic skin lesions over the chest and bilateral arms 30 days after receiving the second vaccine dose, thus indicating a positive correlation between increased dosage and disease progression.

The limitation of our study was the time constraints that hindered detailed examination and analysis.

As COVID-19 immunizations and related side effects are new to the medical field, dermatologists play a crucial role in diagnosing COVID-19 vaccination-related cutaneous sequelae. Our case is relevant for further understanding this relationship, highlighting that additional doses of the Pfizer vaccine contribute to the LP disease progression. Therefore, this should be addressed by institutions administering COVID-19 vaccinations. We suggest further research investigating the concurrent LP onset and multisystem COVID-19 vaccine-related problems due to insufficient current literature, especially regarding COVID-19 vaccination complications on the cutaneous system and other involved systems.

## Data Availability

All data related to the article are available upon request from corresponding authors.
